# Cholera Outbreak in Dadaab Refugee Camp, Kenya — November 2015–June 2016

**DOI:** 10.15585/mmwr.mm6734a4

**Published:** 2018-08-31

**Authors:** Qabale Golicha, Sharmila Shetty, Orkhan Nasiblov, Abubakar Hussein, Eliud Wainaina, Mark Obonyo, Daniel Macharia, Raymond N. Musyoka, Hussein Abdille, Maurice Ope, Rachael Joseph, Willy Kabugi, John Kiogora, Munawwar Said, Waqo Boru, Tura Galgalo, Sara A. Lowther, Bonventure Juma, Robert Mugoh, Newton Wamola, Clayton Onyango, Zeinab Gura, Marc-Alain Widdowson, Kevin M. DeCock, John W. Burton

**Affiliations:** ^1^Kenya Field Epidemiology and Laboratory Training Program, Ministry of Health, Nairobi, Kenya; ^2^Division of Global Health Protection, Center for Global Health, CDC; ^3^United Nations High Commissioner for Refugees, Nairobi, Kenya; ^4^CDC-Kenya, Nairobi;^ 5^Division of Global Migration and Quarantine, National Center for Emerging and Zoonotic Infectious Diseases, CDC; ^6^International Rescue Committee, Nairobi, Kenya;^ 7^Kenya Medical Research Institute, Nairobi, Kenya.

## Abstract

Dadaab Refugee camp in Garissa County, Kenya, hosts nearly 340,000 refugees in five subcamps (Dagahaley, Hagadera, Ifo, Ifo2, and Kambioos) (*1*). On November 18 and 19, 2015, during an ongoing national cholera outbreak (*2*), two camp residents were evaluated for acute watery diarrhea (three or more stools in ≤24 hours); *Vibrio cholerae* serogroup O1 serotype Ogawa was isolated from stool specimens collected from both patients. Within 1 week of the report of index cases, an additional 45 cases of acute watery diarrhea were reported. The United Nations High Commissioner for Refugees and their health-sector partners coordinated the cholera response, community outreach and water, sanitation, and hygiene (WASH) activities; Médecins Sans Frontiéres and the International Rescue Committee were involved in management of cholera treatment centers; CDC performed laboratory confirmation of cases and undertook GIS mapping and postoutbreak response assessment; and the Garissa County Government and the Kenya Ministry of Health conducted a case-control study. To prevent future cholera outbreaks, improvements to WASH and enhanced disease surveillance systems in Dadaab camp and the surrounding area are needed.

## Investigation and Findings

**Case ascertainment.** A suspected cholera case was defined as the occurrence of acute watery diarrhea in any person aged ≥2 years seen at a camp health facility on or after November 18, 2015, or in a child aged <2 years who was epidemiologically linked to a confirmed cholera case. Stool specimens were collected from one of every 2–3 patients with suspected cholera and tested using standard microbiological methods*; cholera isolates were tested for antimicrobial resistance by disc-diffusion. Suspected cases with a stool culture positive for *V. cholerae* were considered to be laboratory-confirmed (*3*). Demographic and clinical data were recorded for all suspected and confirmed cases. Characteristics of cholera cases were described, and case fatality rates calculated. Geographic information system software was used to map the calculated cumulative attack rates by age, sex, and residential block in each of the five subcamps. Spatial clustering of cholera cases by block, adjusted to the block’s population density, was evaluated using the software’s Average Nearest Neighbor function (*4*), which indicated clustering in some blocks.

During November 18, 2015–June 6, 2016, a total of 1,797 cases of cholera, including 1,548 suspected and 249 confirmed, were reported among the camp’s 348,781 residents (Figure); 20 cases that occurred in persons from the host community and were treated in the camp health facilities are included. Males accounted for 904 (51%) cases. The overall attack rate was 5.1 per 1,000 residents, with the highest attack rate occurring in children aged 2–4 years (16.9); attack rates varied by subcamp (Table 1). Fourteen deaths were reported (case fatality rate = 0.79%).

**FIGURE:**
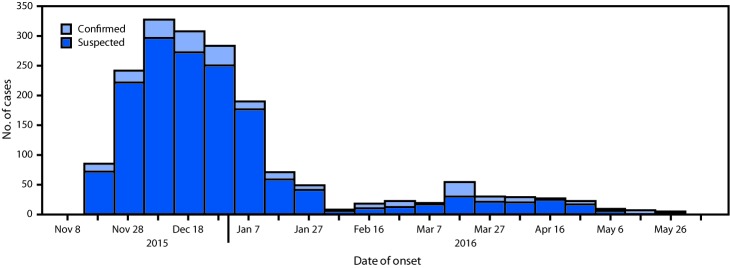
Suspected and confirmed cholera cases (N = 1,797), by week of illness onset — Dadaab refugee camp, Kenya, November 18, 2015–June 6, 2016

**TABLE 1:** Cholera attack rate and case fatality rate, by age and subcamp — Dadaab refugee camp, Kenya, November 18, 2015–June 6, 2016

Subcamp/ Age group	Total population	No. of cases*	Attack rate (per 1,000)	No. of deaths (CFR %)
**Overall**	348,781	1,777	5.1	14 (0.79)
**Subcamp**				
Hagadera	106,925	860	8.0	6 (0.70)
Dagahaley	87,617	678	7.7	5 (0.73)
Kambioos	19,612	103	5.3	2 (1.94)
Ifo2	49,814	83	1.7	1 (1.20)
Ifo	84,813	53	0.6	0 (—)
**Age group (yrs)**				
2–4	32,882^†^	555	16.9	6 (1.08)
5–14	109,808	550	5.0	3 (0.55)
15–24	75,430	253	3.4	0 (—)
≥25	108,739	420	3.9	5 (1.19)

**Abbreviation:** CFR = case fatality rate.

* Twenty cholera cases that occurred in the host community are not included.

^†^ Estimated based on the assumption that among 54,804 children aged 0–4 years, 60% were aged 2–4 years.

After identification of the two index cases on November 18 and 19, the outbreak quickly spread in the subcamps of Hagadera (attack rate = 8.0 per 1,000; peak = December 18–27), Dagahaley (7.7; November 28–December 7), and Kambioos (5.3; December 28–January 6) (Table 1); among 252 residential blocks in these three subcamps, 195 (77%) reported at least one case. Fewer cases were reported in Ifo (attack rate = 0.6 per 1,000) and Ifo2 (1.7), where only 10%–30% of residential blocks reported at least one case. Incidence among affected residential blocks ranged from 0.1% to 20%; spatial clustering of cases occurred in all the subcamps within the residential blocks (p<0.01).

**Identification of risk factors.** In December 2015, the Kenya Ministry of Health conducted a case-control study in the subcamps most affected (Dagahaley and Hagadera) to identify risk factors for cholera. Persons with suspected or confirmed cholera (one per household) clinically evaluated before December 31, 2015, were eligible for inclusion. Eligible controls were Dagahaley or Hagadera residents aged ≥2 years with no history of acute watery diarrhea during the same period. Each case-patient was frequency matched to two controls by subcamp and age group (2–4 years, 5–14 years, 15–24 years, and ≥25 years). A standardized questionnaire was developed that adopted some questions from previous efforts, and it was administered to case-patients and controls (or their caregivers) to collect demographic and exposure information. Partially adjusted odds ratios (ORs) and 95% confidence intervals (CIs) were calculated. Unconditional logistic regression using stepwise forward selection was used for building a multivariate model. Independent variables with p*-*values ≤0.2 in univariate analysis were considered for inclusion. Adjusted ORs (aORs) and 95% CIs were calculated from the final multivariate model.

From a calculated sample size of 38 cases and 76 controls, 32 case-patients and 64 controls were enrolled in the case-control study (Table 2). Identified risk factors for suspected or confirmed cholera included observation by interviewer of 1) human fecal and solid waste in a compound, 2) soiled communal latrines or self-reported open defecation, 3) swimming in rainwater pools, 4) sharing of food from a common plate, and 5) reported sharing of a latrine with someone with diarrhea. Always washing hands with soap and water after using a latrine and household latrine ownership were protective. Living in a compound with visible human and solid waste (aOR = 7.7; 95% CI = 2.0–30.0), self-reported open defecation (13.0; 3.0–61.0), and sharing food on a common plate (5.9; 1.5–23.0) remained significant in the final multivariate model. No evidence of disease clustering by ethnic background or geographic origin was found.

**TABLE 2:** Reported exposures among 32 cholera case-patients and 64 controls during a cholera outbreak — Dadaab refugee camp, Kenya, December 2015

Exposure*	No. reporting exposure (%)		Partially adjusted OR (95% CI)^†^	Adjusted OR (95% CI)^†^
	Cases (N = 32)	Controls (N = 64)		
Use of soiled communal latrine	13 (41)	3 (4.7)	14 (3.6–54.0)	—^§^
Visible solid and human waste in compound	15 (47)	5 (7.8)	10 (3.3–32.0)	7.7 (2.0–30.0)
Swimming in rainwater pools	6 (19)	2 (3.1)	7.2 (1.4–38.0)	—^§^
Sharing latrine with a person with diarrhea	11 (34)	6 (9.4)	5.1 (1.7–15.0)	—^§^
Practicing open defecation	23 (72)	23 (36)	4.5 (1.8–12.0)	13.0 (3.0–61.0)
Sharing food from a common plate	32(100)	42(66)	3.4 (1.5–9.9)	5.9 (1.5–23.0)
Always washing hands with soap and water after using latrine	16 (50)	52 (81)	0.3 (0.1–0.8)	—^§^
Owning household latrine	19 (59)	53 (83)	0.3 (0.1–0.8)	—^§^

**Abbreviations:** CI = confidence interval; OR = odds ratio.

* All participants used communal piped water from tap stands; levels of free residual chlorine and fecal coliforms unknown.

^†^ Unconditional large sample logistic regression. All partially adjusted models included age and residence. Final ORs are adjusted for age, residence, visible solid and human waste in compound, practicing open defecation, and sharing food from a common plate.

**^§^** Adjusted OR not calculated because p*-*value >0.05 for partially adjusted bivariate association.

**Assessment of outbreak control measures.** In late January 2016, as the outbreak waned, CDC and the United Nations High Commissioner for Refugees conducted site visits in four subcamps to assess outbreak control measures. Residual chlorine levels were below outbreak standards in various water sources, including tap stands (outbreak standard = 1.0 mg/L) and households (outbreak standard = 0.5 mg/L), and handwashing facilities in schools, markets, and eateries were insufficient. Pools of stagnant water where children played were observed near affected residential blocks in Dagahaley and Ifo2 subcamps. Although the average number of persons per latrine in Dadaab met the international standard for refugee camps (one latrine per 20 persons) (*5*,*6*), in some subcamps, up to 60 persons were observed to be sharing one latrine. In addition, at the outbreak onset in November 2015, only 168 community health workers were in the camp (approximately one per 2,000 residents), one quarter of the internationally recommended standard of one per 500 residents (*5*). Some households anecdotally reported cases of cholera in multiple household members, although this information was not systematically collected.

**Assessment of antibiotic susceptibility of cholera isolates.** *V. cholerae* serogroup O1, serotype Ogawa was isolated from 312 (39%) of 791 stool specimens. All isolates were sensitive to tetracycline, ceftriaxone, cotrimoxazole, gentamycin, and chloramphenicol; 97% were sensitive to ciprofloxacin. All isolates had intermediate sensitivity to erythromycin and were resistant to furazolidone and nalidixic acid.

## Public Health Response

Cholera treatment centers were established by Médecins Sans Frontiéres and the International Rescue Committee, and active surveillance for cases of acute watery diarrhea was enhanced. A health promotion and hygiene campaign was conducted, primarily through mobilization of community health workers (from 168 during the first few weeks of the outbreak to 286) and hygiene promoters and use of media networks, especially radio. Frequent coordination meetings were held among stakeholders to provide updates and revise recommendations. Water from boreholes was chlorinated, soap was distributed, and bedding and latrine disinfection in affected households was carried out. WASH partners were advised to maintain adequate chlorination levels, install additional tap stands and latrines (especially in unofficial settlement areas), and install additional handwashing facilities in schools, eateries, and marketplaces.

## Discussion

Cholera is an acute diarrheal illness caused by the toxin secreted during infection with *V. cholerae* bacterium after ingestion of contaminated food or water. The infection is frequently mild or asymptomatic; however, approximately 5%–10% of infected persons develop severe disease and profuse watery diarrhea. Without prompt treatment, persons with severe disease can die within hours (*7*). Cholera outbreaks can spread rapidly in densely populated settings such as refugee camps (*8*). Rapid detection and control of cholera outbreaks is a goal for the implementation of the World Health Organization’s International Health Regulations and for global health security.

This cholera outbreak is the largest reported in Dadaab refugee camp since its establishment in 1992. Existing laboratory capacity and effective disease surveillance in the camp facilitated early detection and eventual control of the outbreak. Antimicrobial susceptibility testing confirmed that *V. cholerae* circulating in the camp was susceptible to antibiotics used in treating patients with severe cholera (*9*).

All subcamps were affected, but the highest rates occurred in the two largest subcamps. These two areas had poorer water drainage and were in proximity to food markets where persons from areas with active cholera transmission interact. These factors might have contributed to the large number of cases and high attack rates in these areas. Furthermore, in these subcamps, large numbers of housing structures were outside the official boundaries of the camp, in areas that had poorer water and sanitation infrastructure. Inadequate residual chlorine levels in drinking water and the presence of standing bodies of water used for swimming and bathing suggest possible waterborne transmission routes. Reported infection of multiple household members and spatial case clustering among residential blocks suggest household transmission or common exposures to a contaminated food or water source.

Limited promotion of hygiene messaging during the early weeks of the outbreak could also have increased vulnerability to cholera transmission. Sustained and intensive hygiene promotion and WASH interventions in the most affected blocks were also recommended.

The findings in this report are subject to at least two limitations. First, camp insecurity affected case finding and study enrollment, precluding achieving the calculated sample size for the case-control study. Second, other cases of acute watery diarrhea might have been included as cases of suspected cholera, reducing the power of the study.

The last reported cholera case in the camp occurred on June 6, 2016, and the outbreak was declared over on June 21, 2016. Thereafter, two small clusters of cases not associated with traveling outside the camp or contact with visitors outside the camp were reported in July (seven cases) and August (five cases) 2016. During April–August 2017, a cholera outbreak involving 511 cases occurred after flooding that destroyed approximately 9,000 latrines. In September 2017, four cases were reported, and during October–December 2017, 109 cases were reported. Both outbreaks were controlled immediately with no deaths. Improvements to WASH and disease surveillance are needed to prevent future outbreaks.

**Summary**

**What is already known about this topic?**

Cholera, caused by infection with the bacterium *Vibrio cholerae* through ingestion of contaminated food or water, can spread rapidly in densely populated settings such as refugee camps.

**What is added by this report?**

During November 18, 2015–June 6, 2016, the largest cholera outbreak (1,797 cases; attack rate 5.1 per 1,000) in the history of Dadaab refugee camp in Kenya occurred. Significant risk factors included living in a compound where open defecation, visible human and solid waste, and eating from a shared plate were common. Chlorine levels in water were below standard, and handwashing facilities were insufficient.

**What are the implications for public health practice?**

Improvements to water and sanitation, expansion of capacity for community outreach, and enhanced camp security and disease surveillance systems in Dadaab camp and the surrounding area are urgently needed.
